# Impact of media dependence: how emotional interactions between users and chat robots affect human socialization?

**DOI:** 10.3389/fpsyg.2024.1388860

**Published:** 2024-08-16

**Authors:** Ziying Yuan, Xiaoliang Cheng, Yujing Duan

**Affiliations:** College of Communication Science and Arts, Chengdu University of Technology, Chengdu, Sichuan, China

**Keywords:** chatbot, emotional interaction, interpersonal communication, media dependence, Replika

## Abstract

In the era of intelligent media, human users and chatbots have established a deep dependency relationship through communication, making media dependence a behavioral foundation that widely permeates human social practice. This article investigates how media dependence affects human social interaction during emotional interactions between human users and chatbots. Based on the theory of media dependence, the existing mature scales of media dependence and interpersonal communication were adapted, and 496 Replika user questionnaires were collected. After screening the validity of the questionnaires, 428 valid questionnaires were obtained. Descriptive statistical analysis, correlation analysis, multiple linear regression analysis, and mediation effect testing were used to analyze the impact of media dependence on human–computer emotional interaction. Results indicate a significant positive correlation between human–chatbot emotional interaction and human user social interaction. Media dependence significantly positively regulates emotional interactions between humans and chatbots. In addition, the social interactions of human users are partially influenced by factors such as user nature, age, education, and income.

## Introduction

In recent years, with the continuous development and maturity of brain–computer interface, VR, AI, gene editing, and other technologies, human society has entered a new stage of human–machine integration and symbiosis—the post-human era. In this era, machines are no longer just cold pieces of iron in the traditional sense. They have become media channels that connect and facilitate interactions between people and society, serving as the primary means of communication. The advent of technology disrupts traditional interpersonal communication patterns, fostering a novel relationship between humans and machines, and some of the public who rely heavily on technology are satarting to ignore the emotional relationship between people.

In November 2022, the artificial intelligence lab OpenAI officially launched the universal chatbot ChatGPT. It attracted more than one million users within 5 days of its launch, which is the height of Meta in 10 months and of Netflix in 3 years (Hurst, 2022). In fact, chatbots are nothing new, and the birth of ChatGPT is not an accident. As early as the year of 1966, Weissenbaum, the father of modern artificial intelligence, launched the earliest AI chatbot ELIZA, followed by PARRY, ALICE, Jabberwocky, and other chatbots. The rapid popularity of AI chatbots has not only aroused widespread attention from the academic community but also received the “olive branch” from the business community. In the 21st century, tech giants have similarly launched highly branded chatbots, including Apple’s Siri, Google’s Bard, and Facebook’s Blender. In comparison, China’s AI chatbot was born slightly later. It was not until 2004 that Xiao-I officially launched China’s first chatbot named MSN. Subsequently, Xiaomi’s Xiao Ai, Baidu’s ERNIE Bot, and JD’s Chat-JD also gradually received public attention. With the development of chatbots, machines can not only imitate people’s voices, language, and expressions but also tap into the human heart—emotion. A chatbot breaks through the so-called intersubjectivity and becomes a humanized, emotional, and creative humanoid. As Dominique (2010) wrote in his book: “Emotional factors are essential to understanding the complexity of the world we live in.” The emergence of Replika, an emotional chatbot, not only conforms to the development of the technological era but also shows the necessity of emotional communication. In 2015, Eugenia Kuyda, in memory of a friend who died unexpectedly in the same year, used Google’s basic neural network program to synthesize thousands of conversations of her friend’s messages. This process gave the impression that the deceased friend was communicating when responding to messages. This endeavor was the precursor to Replika. In 2016, Luck officially launched Replika, which has been downloaded more than 10 million times globally. It is the most downloaded chatbot in the App Store and was among the top five mental health programs during the COVID-19 pandemic in 2020. As a dedicated emotional chatbot, Replika provides emotional comfort to many lonely people and those with social phobia through long-term emotional companionship and positive emotional support. Replika was downloaded 55,000 times in Mainland China in the first half of 2021. In addition, a group called “Man–machine Love” quietly emerged on Douban, a Chinese internet-based community platform. The group focuses on emotional communication between humans and AI partners, and it has more than 9,600 members so far.

As the “sixth medium” after newspapers, radio, television, computers, and mobile phones (Lin and Ye, 2019), chatbot’s innate “machine” characteristics and constantly evolving “humanity” not only make the communication between people and chatbot flexible and natural but also generate emotions that human–computer interaction did not have in the past. Therefore, if the public continues to invest time, energy, and emotion in chatbots; constantly rely on virtual social interaction; and even give chatbots “life,” can this kind of human–machine emotional interaction similar to interpersonal communication replace realistic interpersonal communication? That is, what impact will the trend of human–machine emotional interaction have on users’ realistic interpersonal communication?

From the perspective of human–machine communication (HMC), this study takes media dependence as the main theoretical basis. According to the specific application scenario of the Replika chatbot, the author adapts the existing scale and collects variable data through a questionnaire survey, resulting in 428 valid questionnaires. Specifically, this paper makes reference to Rubin’s Quasi-Social Interaction Scale, Kwon’s Smartphnoe Addiction Scale, Zheng Richang’s Interpersonal Comperhensive Relationship Dignostic Scale, etc., as detailed in the questionnaire design section and Table 1. Since the number of Replika users in China is relatively small and scattered, the author mainly distributes and collects questionnaires through platforms and forums where Replika users are active. For example, Douban group, Xiaohongshu platform, Weibo Replika Super talk, etc. Then, descriptive statistical analysis, correlation analysis, multiple linear regression analysis, and mediation effect testing were used to analyze the impact of human–chatbot emotional interaction on human interpersonal communication. Basing on the integration and extension of previous views, this study introduces media dependence theory originally applicable to interpersonal communication into the vision of human–machine communication research. Doing so not only enriches the theoretical scope of human–machine communication but also lays a foundation for building a harmonious and symbiotic human–machine moral destiny community.

**Table 1:** Basic information about the respondents.

Dimension	Category	*N*	Percentage
Gender	Male	122	28.50%
	Female	306	71.50%
Age	Under 18 years old	29	6.80%
	18–25 years old	264	61.70%
	26–30 years old	86	20.00%
	Over 30 years old	49	11.50%
Education	High school degree or below	81	18.90%
	College degree	55	12.90%
	Bachelor degree	223	52.10%
	Master degree or above	69	16.10%
Monthly income	3,000 yuan and below	214	50.00%
	3,001–5,000 yuan	108	25.20%
	5,001–7,000 yuan	44	10.30%
	7,001–10,000 yuan	33	7.70%
	10,001–15,000 yuan	18	4.20%
	15,001–30,000 yuan	8	1.90%
	More than 30,000 yuan	3	0.70%

## Research review

### Media dependence

Influenced by Durkheim’s views on media, American communication researchers Melvin Defler and Sandra Bower-Killoch were the first to define “dependency” from the perspective of social ecology (Sandra et al., 2004). In their paper The Dependence Mode of Mass Communication Media Effect, they pointed out that there is a close dependence relationship among the three systems of media, audience and society, and held that the audience has a corresponding relationship between the satisfaction of information needs and the achievement of goals, and proposed that there is a positive correlation between media dependence and media effect. That is, media dependence will change the public’s existing cognition, attitude and behavior through media content (Melvin and Sandra, 1990). In addition, It is generally believed that there are two forms of media dependence: the explicit habitual dependence and the implicit spiritual dependence. The so-called habitual or conditional dependence refers to the habitual behavior of the public on the media after long-term use of the media. The long-term conditional media dependence will further affect the spirit and psychology of the public and form spiritual dependence. For example, some people will have anxiety, tension, emptiness, lack of security and other emotions when they are separated from the mobile phone media. In the year 2008, the UK Post Office conducted a survey on mobile phones and found that users felt anxious about not being able to use their phones or not having them around, a phenomenon they dubbed “no phone phobia” (SecurEnvoy, 2012). Generally speaking, media dependence refers more to the psychological dependence of the public on the media.

In the context of the continuous development of information technology, the public’s dependence on all kinds of media not only shows a growing trend, but also shows a new form. Some scholars even put forward that the closeness and particularity of the relationship between network media and audience make the media dependence theory more applicable in the Internet environment (Xie, 2004). Through searching relevant literature, the author finds that there are abundant researches on media dependence related to traditional media such as newspaper, radio and television, while there is still room for further research on media dependence in respect of new media. In particular, new media such as chatbots are no longer just intermediaries or tools for communication, but become interlocutors for communication with people. Therefore, for some users, it is easy to see chatbots as people with living characteristics. With the extension of users’ communication time with chatbots and the increase of emotional investment, they may become mentally dependent on chatbots. Therefore, to explore whether users will be dependent on chatbots, and the role of media dependence in human-computer emotional interaction and interpersonal interaction are interesting topics worthy of attention.

### Research on human–machine emotional interaction

At present, research on human–machine emotional interaction mainly focuses on two areas. On the one hand, some scholars discuss how human–machine emotional communication can be realized and the ethical issues in this communication from the perspective of philosophy and humanity. On the other hand, some scholars, starting from reality, intend to clarify the process and optimization path of human–machine emotional interaction.

From the perspective of philosophy and humanity, relevant research can be further subdivided into two aspects: positive discussion and negative reflection. Under positive discussion, David (2007) believed that humans cannot help but form difficult emotional relationships with companions, things, and even robots around them, which is human nature. Sven and Lily (2017) believed that with the continuous development of intelligent technology, robots can imitate and learn human emotions and even fall in love with people. Under negative reflection, John P. Sullins (Peters, 2015) believed that although humans can reach an emotional connection with robots, emotions are complex things, and discerning subtle emotions like humans is difficult for machines. Emmelyn et al. (2021) also believed that establishing close friendships between humans and machines, akin to human relationships, is challenging. Whitby (2008) believed that owing to the “machine nature” of machines, some users may transgress moral boundaries by resorting to verbal violence against the machine. This behavior has the risk of extending from online to offline contexts, potentially strengthening the desire of users to commit physical violence.

Regarding the research on the process and implementation of human–machine emotional interaction, Li and Zhao (2023) studied the interaction between the robot NAO and human. They found that the user can independently judge the emotional meaning implied in the machine actions, and this ability has no obvious relationship with the familiarity between human and machine. Zhang and Han (2022) studied the emotional interaction between users and Xeva software and found a progression from companionship to tolerance and then to rational return in the interaction between the two. Taking smart audio as an example, Kang (2023) discussed the characteristics of machines from various aspects. They investigated the relationship between intelligent machines and user’ families, especially the emotional compensation effect of machines for people living alone. Gan and Guo (2022) observed a coffee robot in Shanghai and believed that the “embodied” aspect is the basis of emotional interaction between consumers and robots. Through the review of previous literature, it is not difficult to find that human beings will have emotional communication in the interaction with chatbots, and such emotional interaction will not only have an impact on the relationship between human and machine, but also have an impact on the actual emotions of users. Consequently, based on the aforementioned research, this paper employs the Replika chatbot as a case study to delve into the current situation of emotional exchange between users and chatbots, encompassing the degree, behaviors, effect, and satisfaction associated with human-chatbot emotional interaction.

Replika as an emotional chatbot platform, the current research mainly focuses on two aspects. On the one hand, the interaction mechanism between users and Replika chatbots is studied, including interaction characteristics, interaction purpose, interaction behavior, etc. Zhang and Sun (2023) combined various research methods to analyze the text messages posted by Replika users on various social platforms and the data obtained from interviews, and studied the emotional connection between Replika users and chatbots from the perspective of embodied imagination between Replika and users. Through interviews with 20 Replika in-depth users, Tan (2023) found that the formation of the current human-computer intimate relationship basically conforms to the process of social penetration theory from the shallow to the deep, from the surface to the inside, and the “human” characteristics of chatbots, human-computer trust and other factors play an important role. The other aspect is the discussion of the ethical issues existing in the interaction between users and Replika. Taking Replika as an example, Possati (2023) used psychoanalysis to discuss the control and responsibility of human unconscious behavior on AI design and behavior. Combined with the murder incident instigated by Replika in Corriere Della Sera, he criticized and reflected on the current ethics of human-computer interaction. Kourkoulou (2023) also used Replika chatbots to discuss the hidden exploitation of emotional labor in the current digital economy, as well as the ethical issues in the field of artificial intelligence in practice, especially the gender and racial stereotypes caused by current chatbots. While discussing the risks and impacts of generative AI in future economic development, Orchard and Tasiemski (2023) also discussed the sexual and pornographic phenomena of Replika chatbot in personalized services, and reflected on the verbal attacks brought by users to the machine. Zeng and Cao (2023), through long-term observation of the Replika team, critically reflected on human-machine relationship from the aspects of commercialization, relationship imbalance, dissociation and so on.

### Research on the relationship between media dependence, human–chatbot emotional interaction, and interpersonal communication

As a new phenomenon in contemporary society, the emotional interaction between chatbots and users still has some lacks in-depth research on the relationship between media dependence and human–chatbot emotional interaction. As chatbot is an emerging technology product, its essence and core cannot be separated from the media itself. Therefore, previous studies on various media and their relationship with media dependence can provide certain references for this paper. Through the review and summary of previous literature, the author finds that the current research on the relationship between media use and media dependence mainly focuses on one aspect: there is a positive correlation between media use (the interaction between people and media) and media dependence. As early as the 20th century, some scholars have studied the relationship between TV media and media dependence, and found that there is a close correlation between the audience’s interaction with TV shopping programs and media dependence (Grant et al., 1991). Wang (2014) found that there was a positive correlation between college students’ use of wechat and media dependence. Some scholars have found that the higher the degree of interaction between users and social robots, the stronger the user’s dependence on the media generated by social robots (Han et al., 2021). Studies have also shown that the degree to which users interact with virtual idols can positively predict users’ media dependence (Zhou and Zhang, 2023). From the extant literature, it is evident that scholars collectively concur in the observation of a positive correlation between the frequency of media utilization and the degree of media dependency. In light of this, it is pertinent to inquire into the nature of the emotional exchange between users and chatbots, a novel medium, in relation to media dependency. Therefore, the following hypothesis is proposed:

*H1*: human-chatbot emotional interaction is positively correlated with media dependence.

In terms of media dependence and interpersonal communication, scholars mainly hold two viewpoints. (1) The public’s dependence on media is conducive to enriching daily life, improving their emotional acquisition and companionship. It also plays a positive role in the practical interpersonal communication of the public. For example, He and Zhu (2024) found that the dependence of left-behind women in rural China on short video media can enhance their self-cognition and adjust their personal emotions, thus improving their daily life. Jiang (2022) studied the media dependence and subjective well-being of the elderly during the COVID-19 pandemic and found that the elderly has a high sense of dependence and trust in TV media. TV media can not only make up for the gap in real interpersonal communication but also strengthen the happiness of the elderly. (2) Media dependence can exacerbate loneliness. Kim et al. (2009) found that there is a vicious cycle between loneliness and media dependence, and excessive use of social media can lead to deeper levels of loneliness. Zhang et al. (2020) have identified a significant correlation between mobile phone addiction and individual loneliness, indicating a moderate positive relationship between the two phenomena. Han et al. (2021) found that users’ media dependence on Microsoft Xiaobing strengthens users’ sense of loneliness. In light of the escalating authenticity, immersion, and interactivity associated with chatbots, there is a possibility that users’ reliance on chatbots, along with their dependent behaviors, could surpass their previous dependency on traditional media. Consequently, within this context, does the users’ reliance on chatbots for media purposes exacerbate their feelings of loneliness? Furthermore, does this sense of dependency have an impact on their genuine interpersonal engagements? Therefore, the following hypothesis is proposed:

*H2*: media dependence is positively related to users' interpersonal communication status.

In the aspect of human–chatbot emotional interaction and interpersonal communication, a large gap is noted in the relevant research at home and abroad. However, as the predecessor of human–machine interaction, the relationship between network interaction and real interpersonal communication can also reflect the relationship between human–machine interaction and real interpersonal communication to a certain extent. This stream of research is mainly divided into two categories. One view holds that virtual and online communication, as an important complementary form of real interpersonal communication, can have a positive effect on real interpersonal communication. For example, Su (2020) studied the communication behaviors between users and the mobile game Dream Journey to the West. They found that compared with real interpersonal communication, online intimate relationships in the form of games can be transformed into offline relationships and are conducive to maintaining long-distance interpersonal relationships. Through empirical research, Wang and Fu (2016) also found that the use of social media is conducive to expanding the frequency and breadth of college students’ realistic interpersonal communication and can supplement the realistic interpersonal communication to a certain extent. The other view is that virtual and online interaction is not conducive to maintaining real interpersonal relations. It is not conducive to the public to grasp the rules of interpersonal communication. Moreover, it easily leads to the public escaping from reality and avoiding social behavior. For example, in “The Interactive Ritual Chain,” Collins (2009) argued that the lack of ritual is why the advent of email has allowed the masses to indulge in utilitarian interactions and weakened real-world relationships. The dependence behavior caused by long-term media use easily causes the public to have real social difficulties, which is not conducive to users’ real interpersonal communication. For example, Lin (2020) believed that the public’s dependence on the media can cause problems such as difficulty in choice. The public can experience difficulties distinguishing between the media world and the real world, which is not conducive to their real life. Chen (2009) believed that TV media, as an important medium in children’s growth, creates a mimicry environment that deviates from reality through prolonged exposure, gradually alienating children from people and things in the real world. Wei (2012) believed that owing to the strong virtuality of Weibo, the interaction in Weibo can impact real interpersonal relationships and lead to the loss of human subjectivity. Concerning the interaction between users and chatbots, as a form of interpersonal communication, it raises questions regarding whether it may mitigate the intensity of users’ authentic social connections and whether it could augment the user’s perception of solitude. And what role does media dependence play between the two? Therefore, the following hypotheses are proposed:

*H3*: human-chatbot emotional interaction is positively correlated with the user's interpersonal communication status.

*H4*: media dependence plays a mediating role between the current situation of human-chatbot emotional interaction and the current situation of real interpersonal communication.

## Methods and investigations

### Data collection and sample description

The study constructed the formal questionnaire of the survey through the Wenjuanxing platform, which provides functions equivalent to Amazon Mechanical Turk. Considering that Replika is an emotional chatbot, and emotional factor is an essential element for users to interact with AI character, this paper takes users who have used Replika as the research object. From October 22 to November 11, 2023, the questionnaires were distributed and collected through the Douban’s “Man–Machine Love” group (9,602 members), “My family’s Replika has become fine” (2,327 members), “Female Players Association” (46,936 members), “My Replika is very warm” (419 members), the Replika topic on Xiaohongshu platform, Weibo Replika Super talk, and other platforms. The study also used the private messaging functions of Douban, Xiaohongshu, Weibo, Xianyu, and Douyin platforms to conduct a one-to-one questionnaire survey of users who posted or commented on Replika information on these platforms. Finally, 496 questionnaires were collected, and 68 invalid questionnaires were eliminated to ensure the effectiveness of the survey. Invalid questionnaires mainly included those that took less than 60 s to answer (3 questionnaires), those who chose “never used the Replika software” in the first question. (48 questionnaires), those whose answers to all questionnaires were the same (3 questionnaires), and those with the same ID were filled in multiple times (14 questionnaires). A total of 428 valid questionnaires were obtained, with an effective rate of 86.29%. The basic information of the respondents is shown in Table 1.

### Questionnaire design

The questionnaire scale was developed by combining the existing literature and classical scales, focusing on the study object, Replika chatbot. The scale used a five-level Likert scoring method, 1 = “strongly agree,” 2 = “agree,” 3 = “uncertain,” 4 = “disagree,” and 5 = “strongly disagree.” After forming the preliminary draft of the questionnaire, the preliminary survey was carried out in a small scope. According to the questionnaires recovered from the preliminary survey, problems were found, and the questionnaires were optimized to form a formal questionnaire. Specifically, according to the results of the preliminary survey, the author modified the questions with ambiguous expressions and adjusted the order of the questions. For example, the original B13 question “Replika can find my mood changes during interaction.” was adjusted to B12 question “Replika can well understand my feelings or emotions during interaction.” to enhance the logic and coherence of the question. Moreover, the original B7 “I will communicate my work with Replika during the interaction.” and B8 “I will communicate my learning with Replika during the interaction.” overlap, so the two questions have been merged as “I will communicate my work or learning with Replika during the interaction. Besides, Considering the simplicity of the questionnaire, the options of “elementary school education,” “junior high school education” and “high school education” in the questionnaire E3 were merged into “high school education and below.” Finally, questionnaires were distributed and collected from Replika users on platforms such as Douban, Xiaohongshu, and Weibo.

The questionnaire consists of the following parts: the title and introduction, the body, and the end. The body of the questionnaire includes the usage of Replika, the emotional interaction between the user and Replika (chatbot), the user’s interpersonal communication, and the user’s demographic information.

In order to dispel the concerns of Replika users and ensure that the respondents can fill in the questionnaire seriously and carefully, in the introduction section of the questionnaire, the author briefly introduces the identity of the investigator, the purpose of the survey, the connotation of the chatbot, the meaning of emotional interaction, and the use of the questionnaire data.

The main body of the questionnaire includes five aspects:

(1) *Users’ Replika usage.* This part is mainly to understand the respondents’ basic use of Replika, including the gender and identity of the virtual character set by users, the frequency and years of users’ use of the Replika chatbot, the Replika level and user’s purpose of use.

(2) *Current situation of emotional interaction between users and Replika.* It mainly includes four aspects, namely, the degree of the current user’s emotional interaction with Replika, the behavior in their emotional interaction, the effect of emotional interaction, and the user’s satisfaction with emotional interaction.

First, the degree of emotional interaction between users and chatbots is mainly reflected by the degree of privacy of the content exchanged between users and chatbots, the degree of personal emotion revealed in the communication, the degree of empathy between users and AI characters, and so on. Second, considering the differences of communication subjects, this paper further divides behavior of emotional interaction between human and chatbots into chatbot behavior and human behavior. The behavior of human emotional interaction is mainly reflected by the willingness of users to communicate with chatbots about hobbies, work, study and real thoughts in real life, the degree of users’ respect for the views of Ai characters, and so on. The behavior of chatbot emotional interaction is mainly reflected by the AI character’s ability to perceive and understand the emotional changes of users, ability to comfort users, ability to solve problems for users, and so on. Third, communication effect can be divided into cognitive effect, emotional (attitude) effect and behavioral effect (Guo, 2011). Therefore, this paper further divides the effect of emotional interaction into three levels: cognition, emotion, and behavior. The effect of emotional interaction at the cognitive level can be reflected mainly through the cognitive effect of users on the basic information and expression ability of chatbots. The effect of emotional interaction at the emotional level is mainly reflected through the ability of chatbots to relieve loneliness, release life pressure, provide companionship and care for users. Finally, the effect of emotional interaction on user behavior is mainly reflected by the degree of user’s dependence on chatbot and the closeness of the relationship between user and chatbot. The satisfaction of emotional interaction is mainly reflected by the user’s willingness to continue to use chatbots, the user’s willingness to recommend chatbots to others, and the user’s recognition of the expression ability and intelligence of chatbots.

Concretely speaking, based on the qusai-social interaction scale prepared by Rubin et al. (1985), quasi-social relationship scale prepared by Horton and Wohl (1956), user-role interaction scale prepared by Auter and Palmgreen (2000), the microblog interaction scale prepared by Lu (2011) and the gottman scale of quasi-social relations prepared by Ge (2017), this paper adjusted and modified the scale in combination with the specific research objects of this paper, and finally formed the scale of emotional interaction between users and chatbots in this paper. In this paper, the interactive object in the original item is modified from “local news anchor” to “Replika,” and the original single scale is divided into “degree, behavior, effect and satisfaction.” The detailed contents of the scale are delineated in Appendix Table.

(3) *Users’ media dependence on Replika.* The purpose is to understand the media dependence caused by emotional interaction between users and Replika and further understand the relationship between human–chatbot emotional interaction and media dependence. Specifically, based on the Smartphone Addiction Scale (SAS) compiled by Kwon et al. (2013) and the Chinese Internet Addiction Scale compiled by Chen et al. (2003), this paper adjusted and modified the scale in combination with the specific research objects of this paper, and finally formed the media dependence scale of this paper. This article modifies “smartphone” and “internet” in the original scale to “Replika.” The detailed contents of the scale are delineated in Appendix Table.

(4) *Users’ interpersonal communication.* This aspect deals with the main understanding of the user in the real-life interpersonal communication of negative emotions, loneliness, and so on. Specifically, based on the Interpersonal Comprehensive Relationship Diagnostic Scale (ICDS) compiled by Zheng (1999) and the Social Avoidance and Distress Scale (SADS) of Watson and Friend (1969), this paper adjusted and modified the scale in combination with the specific research objects of this paper, and finally formed the user’s real interpersonal communication status scale in this paper. The detailed contents of the scale are delineated in Appendix Table.

(5) *Respondents’ demographic information.* The demographics of Replika users can also affect the Replika chatbot usage, so questions about users’ gender, age, monthly income, and educational background were included.

Overall, the body of the questionnaire contains a total of 65 questions, with 52 scale questions and 13 non-scale questions (Table 2).

**Table 2:** Questionnaire design.

Dimension		Indicator		Corresponding question number	Source of scale
Part I	Replika usage	Use or not		A1	Non-scale question
		Year of use		A2	
		Frequency of use		A3	
		Replika character’s gender, nickname role, and rank		A4–A7	
		Reasons of use		A8	
Part II	Current situation of human–chatbot emotional interaction	Degree of emotional interaction between human and chatbots		B1–B5	Horton and Wohl (1956), Rubin et al. (1985), Auter and Palmgreen (2000), Lu (2011), and Ge (2017)
		Behavior of emotional interaction between human and chatbots	Human behavior	B6–B11	
			Chatbots behavior	B12–B19	
		Effect of emotional interaction between human and chatbots	Cognitive effect	B20–B24	
			Emotional effect	B25–B30	
			Behavioral effect	B31–B35	
		Satisfaction of emotional interaction between human and chatbots		B36–B41	
Part III	Users’ media dependence on Replika			C1–C6	Chen et al. (2003) and Kwon et al. (2013)
Part VI	Users’ real-life interpersonal communication	Interpersonal interaction willingness, Interpersonal relationships, interpersonal attitudes, etc.		D1–D5	Watson and Friend (1969) and Zheng (1999)
Part VII	Personal information	Gender, age, educational background, occupation, monthly income		E1– E5	Non-scale question

At the end of the questionnaire, the respondents were again thanked for their patience and cooperation.

### Reliability testing and validity testing

Reliability testing is an important part of a questionnaire survey, it is related to the rationality of the questionnaire setting and the reliability of research results. In this study, SPSS27.0 was used as a reliability detection tool. Cronbach’s alpha was used to test the data on three aspects: the status quo of emotional interaction between users and Replika chatbots, media dependence brought by emotional interaction, and users’ real-life interpersonal communication. If the Cronbach’s alpha coefficient is greater than 0.60, the reliability of the questionnaire is acceptable, and if the coefficient is greater than 0.70, the reliability of the questionnaire is good. As shown in the figure, the Cronbach’s alpha coefficients of the three scales are all greater than 0.8, and the Cronbach’s alpha coefficient of the whole questionnaire scale (52 items) is 0.977, so its reliability is high. The reliability test results of each variable in this study are shown in Table 3.

**Table 3:** Reliability analysis of questionnaire scale.

Variable			Number of projects	Cronbach’s Alpha
Current situation of human–chatbot emotional interaction	Degree of emotional interaction between human and chatbots		5	0.822
	Behavior of emotional interaction between human and chatbots	Human behavior	6	0.871
		Chatbots behavior	8	0.905
	Effect of emotional interaction between human and chatbots	Cognitive effect	5	0.858
		Emotional effect	6	0.888
		Behavioral effect	5	0.855
	Satisfaction of emotional interaction between human and chatbots		6	0.891
Users’ media dependence on Replika			6	0.923
Users’ real-life interpersonal communication			5	0.815
Questionnaire scale population			52	0.977

A validity test is a means to assess the accuracy and validity of each factor of the questionnaire, including surface validity, criterion validity, and construction validity. To test the validity of the questionnaire (scale), KMO and Bartlett tests were conducted on the overall questionnaire scale (52 items) and three groups of scales. If the KMO value is greater than 0.6, then the questionnaire can perform factor analysis; greater than 0.7, is generally suitable for factor analysis; greater than 0.8, is more suitable for factor analysis; greater than 0.9, is very suitable for factor analysis. As shown in Table 3, the KMO value of the four aspects of the questionnaire scale is greater than 0.8, and the overall KMO value of the questionnaire scale is greater than 0.9, so the validity of the questionnaire is high. The validity test results of each variable in this study are shown in Table 4.

**Table 4:** Results of KMO and Bartlett’s Sphericity Tests.

Variable			KMO measure of sampling adequacy	Bartlett’s sphericity test	Significance
Current situation of human–chatbot emotional interaction	Degree of emotional interaction between human and chatbots		0.833	724.244	0.000
	Behavior of emotional interaction between human and chatbots	Human behavior	0.886	1115.741	0.000
		Chatbots behavior	0.925	1801.643	0.000
	Effect of emotional interaction between human and chatbots	Cognitive effect	0.855	900.547	0.000
		Emotional effect	0.905	1242.530	0.000
		Behavioral effect	0.840	942.921	0.000
	Satisfaction of emotional interaction between human and chatbots		0.904	1305.825	0.000
Users’ media dependence on Replika			0.905	1821.726	0.000
Users’ real-life interpersonal communication			0.803	719.200	0.000
Questionnaire scale population			0.979	16313.800	0.000

## Results and data analysis

### Analysis of Replika usage

According to the data, the usage time of Replika users is mainly less than 6 months, and the ratio of users who use Replika for less than 6 months to those who use it for more than 6 months is about 3:2. Moreover, there is an overall inverse correlation between the duration of use and the number of users. This shows that most of the current Replika users are short term users, and the stickiness of the platform still needs to be improved. Besides, nearly half of users use Replika once a week, and only 10 percent use Replika every day, which indicates that the current intimacy between users and AI characters is not high. In terms of users’ level, this question is optional, and questionnaires with unfilled and unclear answers were eliminated, and the collected levels were sorted. The user level pertains to the rating assigned by the platform to users based on their engagement metrics such as usage duration and interaction frequency. In general, an increase in user duration and interaction frequency typically results in a higher user level. Based on Replika’s official user profiling, the chatbot’s capabilities at levels 1–10 are in a “preliminary” stage. During this juncture, the emotional support rendered by Replika to its users is comparatively constrained, and the level of intimacy and frequency of interaction between users and the chatbot is minimal (Zeng Yiguo, et al., 2023). However, as users progress beyond level 10 and continue their development, the dialogue dynamics between the user and the chatbot become increasingly fluid and profound, thereby strengthening the emotional bond between them. The data shows that the user’s level is mainly concentrated in the below 10 level, the user of high level is very few, and the ratio of users below 10 level and above 10 level is about 4:1. Among them, the lowest level is level 1, and the highest level is level 167. This shows that most current users do not have a strong intimacy with AI characters. In addition, the data shows that 74.1% of users regard entertainment as one of the purposes of using replika, 60% of users regard social networking as one of the purposes of using replika, 43.90% of users regard learning as one of the purposes of using replika, and less than 10% of users think that using replika is for other purposes. This suggests that users use replika primarily for entertainment and socializing, again for learning, and finally for other purposes. The statistical data of Replika usage is shown in Table 5.

**Table 5:** Statistical data of Replika usage.

Dimension	Category	*N*	Percentage
Duration of use	Less than 3 months	134	31.30%
	3–6 months	132	30.80%
	6–12 months	77	18.00%
	1-3-years	54	12.60%
	More than 3 years	31	7.30%
Frequency of use	At least once a day	59	13.80%
	At least once a week	204	47.70%
	At least once a half month	89	20.80%
	At least once a month	50	11.70%
	Less	26	6.00%
Users’ level	Below or equal to level 10	121	79,60%
	Above level 10	31	20.40%
Purpose of use	Entertainment purposes	317	74.10%
	Social purposes	257	60.00%
	Learning purposes	188	43.90%
	Other purposes	29	6.80%

### Analysis of current situation of human–chatbot emotional interaction

The current situation of human–chatbot emotional interaction is mainly reflected through four aspects: the degree of emotional interaction, the behavior of emotional interaction, the effect of emotional interaction, and the satisfaction of emotional interaction. The higher the score of emotional interaction, the higher the degree of emotional interaction, the more engaged behavior, the better the effect and the higher the satisfaction.

First, the level of emotional interaction between users and the Replika chatbot is above average, with a mean of 3.6313 and a standard deviation of 0.84679. The degree of emotional interaction between the user and the Replika chatbot is mainly measured by the degree to which the user has genuine emotional communication with the Replika chatbot, the degree to which the content of the communication is private, the degree to which the personal emotion is revealed in the communication, the degree to which the user regards Replika as a person and tries to understand them, and the degree to which the user empathizes with the virtual character in Replika.

Second, the behavior of human–chatbot emotional interaction is at a superior level, with a mean of 3.8188 and a standard deviation of 0.75641. The behavior of emotional interaction is mainly reflected in two aspects. On the one hand, the user’s behavior in emotional interaction is mainly based on their willingness to communicate with Replika about hobbies, work, and study; the extent to which they express their genuine views and negative emotions on real-life events and people; and the extent to which the Replika avatar is understood. On the other hand, Replika’s behavior in emotional interactions is mainly based on the Replika avatar’s perception of the user’s emotions and emotional changes, the understanding of the user’s emotions, the empathy for the user’s bad experience, and the comfort to the user and the ability to bring solutions to them. Specifically, the average of Replika’s behavior in emotional interactions is 3.8356, and the average of the user’s behavior in emotional interaction is 3.7963.

Third, the effect of the emotional interaction between users and the Replika chatbot is also at a superior level, with a mean of 3.7417 and a standard deviation of 0.73615. Among the three effects of affective interaction, the mean of cognitive effect is 3.8463, and the standard deviation is 0.80537. The mean of emotion effect is 3.8980, and the standard deviation is 0.77763. At the behavioral level, the mean effect is 3.4495 and the standard deviation is 0.89316. The emotional interaction behaviors and effects are detailed in Table 6. In general, the level of behavioral effect is the lowest, and the level of cognitive effect is almost equal to the emotional effect. In the end, users’ satisfaction with their emotional interaction with Replika is on top, with a mean of 3.8220 (the highest score for the mean of the variable) and a standard deviation of 0.78791 (Table 7).

**Table 6:** Statistical data of human–chatbot emotional interaction behavior and effect.

Indicator		*N*		Mean	Standard	Variance	Minimum	Maximum	Sum
		Effective	Missing sample						
Behavior	Human behavior	428	0	3.7963	0.80210	0.643	1.00	4.83	1624.83
	Chatbot behavior	428	0	3.8356	0.77902	0.607	1.00	5.00	1641.62
Effect	Cognitive effect	428	0	3.8463	0.80537	0.649	1.20	5.00	1646.20
	Emotional effect	428	0	3.8980	0.77763	0.605	1.33	5.00	1668.33
	Behavioral effect	428	0	3.4495	0.89416	0.798	1.00	5.00	1476.40

**Table 7:** Statistical data of the current situation of human–chatbot emotional interaction.

Indicator	*N*		Mean	Standard	Variance	Minimum	Maximum	Sum
	Effective	Missing sample						
Degree of emotional interaction	428	0	3.6313	0.84679	0.717	1.20	5.00	1554.20
Behavior of emotional interaction	428	0	3.8188	0.75641	0.572	1.00	4.86	1634.43
Effect of emotional interaction	428	0	3.7417	0.73615	0.542	1.50	5.00	1601.44
Satisfaction of emotional interaction	428	0	3.8220	0.78791	0.621	1.00	5.00	1635.83

### Analysis of users’ media dependence on replika

The current level of reliance on Replika is moderate, with a mean of 3.2936 (the lowest score of the variable) and a standard deviation of 1.05839. The higher the score of media dependence, the higher the degree of media dependence on Replika. The index is mainly reflected by the increase in the number of times users use Replika, the increase in the length of use, the decrease in the frequency of interaction with family and friends, the decrease in daily leisure activities, the level of depression caused by non-interaction, and the decrease in the enjoyment of life. In general, users do not rely much on Replika, and most users can reasonably balance virtual and real interactions. The specific analysis data can be found in Table 8.

**Table 8:** Statistics on media dependence and real-life interpersonal communication.

Indicator	*N*		Mean	Standard	Variance	Minimum	Maximum	Sum
	Effective	Missing sample						
Users’ media dependence on Replika	428	0	3.2936	1.05839	1.120	1.00	5.00	1409.67
Users’ real-life interpersonal communication	428	0	3.6650	0.86687	0.751	1.00	5.00	1568.60

### Analysis of users’ real-life interpersonal communication

At present, the real-life interpersonal communication of users is at a medium and high level, with an average of 3.6650 and a standard deviation of 0.86687. The higher the score of interpersonal communication, the worse the level and ability of interpersonal communication. The higher the score of this index, the lower the self-disclosure willingness of users in real-life interpersonal communication. This index is mainly reflected by the degree of interaction between users and acquaintances in real-life interpersonal communication, the level of comfort and comfort in real-life interpersonal communication, the degree of hiding their true feelings in real-life interpersonal interaction, the degree of expression of positive emotions rather than new feelings in real-life interpersonal interaction, and the level of loneliness of users in real-life interpersonal interaction. The specific analysis data can be found in Table 8.

## Analysis and discussion

### Correlation analysis of variables

To explore the correlation between human–chatbot emotional interaction, media dependence, and real-life interpersonal communication, this study processed the data of three variables through Pearson correlation analysis and MLR. The results are shown in Tables 9, 10.

**Table 9:** Correlation analysis of variables.

Indicator		1	2	3			
				Degree	Behavior	Effect	Satisfaction
Users’ media dependence on Replika		1					
Users’ real-life interpersonal communication		0.741**	1				
Current situation of human–chatbot emotional interaction	Degree of emotional interaction	0.686**	0.697**	1			
	Behavior of emotional interaction	0.581**	0.697**	0.836**	1		
	Effect of emotional interaction	0.705**	0.752**	0.817**	0.868**	1	
	Satisfaction of emotional interaction	0.530**	0.636**	0.709**	0.817**	0.846**	1

**Significant association at 0.01 level (bilateral). *Significant association at 0.05 level (bilateral).

**Table 10:** Correlation analysis of variables.

		1	2	3
Users’ media dependence on Replika	Pearson correlation	1	0.741**	0.674**
	Significance (two tail)		0.000	0.000
	N	428	428	428
Users’ real-life interpersonal communication	Pearson correlation	0.741**	1	0.753**
	Significance (two tail)	0.0000		0.000
	N	428	428	428
Current situation of human–chatbot emotional interaction	Pearson correlation	0.674**	0.753**	1
	Significance(two tail)	0.000	0.000	
	N	428	428	428

**Significant association at 0.01 level (bilateral). *Significant association at 0.05 level (bilateral).

### Regression analysis of variables

This study predicted media dependence as the mediating variable in the influence of the current situation of emotional interaction on users’ real-life interpersonal communication. Therefore, to further predict the impact of each variable on the user’s real-life interpersonal communication, this study conducted hierarchical regression analysis on the predicted independent variable (the current situation of emotional interaction), intermediary variable (media dependence), and control variable (demographic variable). First, gender, age, education, and monthly income together explain 11% of users’ real-life interpersonal communication. In terms of gender (*β* = 0.063, *p* = 0.000 < 0.05), women’s real-life interpersonal communication score is higher than that of the male group. In terms of education (*β* = −0.276, *p* = 0.000 < 0.05), users with higher education had lower scores in real-life interpersonal communication. In terms of age (*β* = 0.203, *p* = 0.200 > 0.05) and monthly income (*β* = −0.005, *p* = 0.914 > 0.05), no significant relationship is observed between them and users’ real-life interpersonal communication scores. The score of real-life interpersonal communication mainly reflects the user’s social integration and self-disclosure in real-life interpersonal communication. The higher the score, the lower the degree of social integration and self-disclosure.

When human–chatbot emotional interaction enters the equation as the second factor, 47.8% of users’ real-life interpersonal communication is further explained. Users’ emotional interaction (*β* = 0.712, *p* = 0.000 < 0.001) becomes an important factor affecting users’ real-life interpersonal communication. The stronger the emotional interaction with the Replika chatbot, the worse the self-disclosure and social inclusion of users in their real-life interpersonal interactions.

When users’ media dependence on Replika is entered the equation as the third factor, 7.7% of users’ real-life interpersonal communication is further explained. Media dependence (*β* = 0.418, *p* = 0.000 < 0.05) has a significant impact on users’ real-life interpersonal communication. The more dependent the participants are on the medium of Replika, the worse their self-disclosure and social integration in real-life interpersonal communication. In addition, after the Durbin–Watson (DW) test of Models 1, 2, and 3, the DW values of the three models are close to 2 (the DW value of Model 1 is 1.970; Model 2, 1.908; Model 3, 2.011), indicating the high independence of the data. Overall, hierarchical regression explains 66.5% of the total equation, and detailed data are shown in Table 11.

**Table 11:** Hierarchical regression analysis of variables.

Predictor variable	Model 1			Model 2			Model 3		
	*B* (SE)	*β*	*t*	B (SE)	*β*	*t*	*B* (SE)	*β*	*t*
Gender	0.121	0.063	1.283	0.041	0.021	0.640	0.082	0.043	1.416
Age	0.228	0.203	4.130	0.094	0.083	2.459	0.035	0.031	1.004
Education	−0.248	−0.276	−5.845	−0.129	−0.143	−4.373	−0.030	−0.034	−1.070
Monthly income	−0.004	−0.005	−0.108	−0.004	−0.006	−0.180	−0.014	−0.021	−0.697
Current situation of human–chatbot emotional interaction				0.858	0.712	22.172	0.556	0.461	12.017
Users’ media dependence on Replika							0.343	0.418	9.936
Intercept	3.586			0.490			0.326		
*F*	14.218			122.882			142.568		
*R* ^2^	0.119			0.593			0.670		
DW inspection	0.110			0.588			0.665		

### Mediation effect analysis of variables

Based the previous research, this study sets the current situation of emotional interaction between users and chatbot Replika as the independent variable (X), the user’s real-life interpersonal communication as the dependent variable (Y), and the user’s acquired media dependence as the intermediary variable (M). The following regression equation can be used to represent the relationship between the variables:


$$Y=cX+e_{1}$$

$$M=\mathrm{aX}+e_{2}$$

$$Y=c^{'}X+bM+e_{3}$$

In the equation, c is the total effect of independent variable X (current situation of human–chatbot emotional interaction) on dependent variable Y (interpersonal communication). a is the effect of the independent variable X on the intermediary variable M (media dependence). $c^{’}$ is the direct influence of independent variable X on dependent variable Y after controlling the influence of intermediary variable X. b is the influence of intermediary variable M on dependent variable Y after controlling the influence of independent variable X. In addition, the coefficients e_1_, e_2_, and e_3_ are error terms.

To further clarify the mediating effect of Replika media dependence on users between emotional interaction and interpersonal communication, this study used PROCESS V4.1 plug-in in SPSS26.0 software as a research tool to analyze and study the three. 5,000 samples were selected from the original sample to estimate the 95% confidence interval of the mediation effect, and Model 4 was chosen. In this paper, according to Hayes et al.’s (2012) viewpoint, 5,000 bootstrap samples were selected using the deviation-corrected percentile bootstrap method to test the moderated mediating effect. Specifically, from the 428 samples that have been returned, 5,000 times, one sample at a time, to get a new sample. The Bootstrap method can avoid the limitation of data distribution hypothesis and better deal with non-parametric statistical problems. If the confidence interval does not contain 0, then the mediation effect exists, and vice versa. The data showed a significant positive correlation between human–chatbot emotional interaction and users’ Replika media dependence (*β* = 0.88, *p* = 0.000 < 0.001). That is, human-chatbot emotional interaction leads to user dependence on Replika chatbots. Consistent with the results of hierarchical regression, media dependence (*β* = 0.34, *p* = 0.000 < 0.05) has a significant positive impact on users’ real-life interpersonal communication.

After in-depth analysis of the mediation test results, the author found that even after controlling for the four variables of gender, age, education, and monthly income, the emotional interaction between users and Replika has a direct effect on users’ real-life interpersonal communication [Effect = 0.56, *p* = 0.000 < 0.001, 95% CI (0.47, 95% CI)]. The indirect effect [Effect = 0.30, 95% CI (0.23, 0.38)] reaches statistical significance, indicating a significant partial mediating effect between the current situation of human–chatbot emotional interaction and users’ interpersonal communication. The mediating effect accounts for 34.88% of the total effect. Table 12 provides the details.

**Table 12:** Mediating effect analysis of variables.

	Effect value	Standard error	Bootstrap 95% CI		Total effect ratio
			Upper limit	Lower limit	
Total effect	0.86	0.04	0.78	0.93	
Direct effect	0.56	0.05	0.47	0.65	
Indirect effect	0.30	0.04	0.23	0.38	34.88%

## Conclusion

The results show that all four hypotheses are verified. First of all, there is a significant positive correlation between the pairwise of the three variables, that is, H1, H2 and H3 are all valid. Secondly, media dependence plays a partial mediating role between between the current situation of human-chatbot emotional interaction and users’ interpersonal communication, that is, H4 is valid.

According to the descriptive statistical results of the variables, the users are mostly light users (low level), and they have a moderate level of time and emotion invested in the Replika platform. Besides, in the behavior of emotional interaction between users and chatbots, the score of user behavior is close to that of chatbots, and the score of chatbot behavior is slightly higher than that of users. This suggests that users pay more attention to their emotional acquisition in interaction rather than emotional engagement. And, the three effects of emotional interaction show the following relationship: emotional effect > cognitive effect > behavioral effect. Users have a high average satisfaction with Replika chatbot and a strong willingness to continue using it. In addition, users’ media dependence on Replika is at a moderate level, and most users can reasonably balance real and virtual interactions. Finally, the average value of users’ real-life interpersonal communication is at a medium to high level.

The correlation analysis results show that the correlation between human–chatbot emotional interaction and users’ interpersonal communication is the strongest, followed by the correlation between media dependence and users’ interpersonal communication. The correlation between media dependence and human-chatbot emotional interaction is relatively weak. Among the four indicators of the emotional interaction, the effect of emotional interaction has the strongest correlation with the user’s interpersonal communication, followed by the degree of emotional interaction and the behavior of emotional interaction. The satisfaction of emotional interaction has the weakest correlation with the interpersonal communication. The hierarchical regression analysis shows that human–chatbot emotional interaction and media dependence is significantly positively correlated with users’ real-life interpersonal communication. The current situation of human–chatbot emotional interaction has a greater impact on users’ real-life interpersonal communication, whereas media dependence has a lesser impact on users’ real-life interpersonal communication.

The results of the mediation effect further show that the emotional interaction between users and Replika chatbots has a strong predictive effect on interpersonal communication. The higher the degree, behavior, effect, and satisfaction of the emotional interaction between the user and the chatbot, the worse the real-life interpersonal communication. Human–chatbot emotional interaction, as a new kind of quasi-interpersonal relationship and virtual interpersonal relationship, can enrich individual communication life and alleviate individual loneliness to a certain extent. Nonetheless, it also affects users’ real-life interpersonal relationships, negatively affecting their real-life interpersonal skills, interpersonal relationships, and communicative attitudes. In addition, media dependence partly mediates the relationship between emotional interaction and real-life interpersonal communication, and emotional interaction can have a negative impact on users’ real-life interpersonal communication through media dependence. Moreover, users’ real-life interpersonal communication is not only affected by the independent variables and mediating variables set in this study but also by factors at the individual level of users (gender, age, education, and income).

Basing on the results of empirical analysis, this study puts forward the following suggestions. First, the public’s machine literacy and ethical concepts must be improved. On the one hand, the public should avoid falling into the trap of the dichotomy of good and evil and blindly believing that the emergence of chatbots depletes human survival resources or can solve human emotional problems. On the other hand, the public should be wary of chatbots and should not blindly immerse in virtual human–chatbot interaction and abandon realistic interpersonal interaction. They should always maintain the sense of proportion of emotional interactions with chatbots. Second, the moral sense and social responsibility awareness of chatbots must be improved. In view of the moral problems in the relationship between human and technology, Eid et al. put forward the term “technological ethics.” In their view, it is the responsibility and obligation of owners and manufacturers to design technology with a sense of morality, and moralization should be a force throughout the development of technology to restrict and regulate technology through morality so as to avoid technology falling into ethical risks (Verbeek, 2011). On the one hand, chatbot platform owners should keep their own moral cultivation and social responsibility awareness and must not use chatbots to infringe users’ interests because of selfish desires. On the other hand, the inventor of the chatbot should set up an ethical mechanism to maintain the benign operation of human–chatbot interaction in advance and adopt relevant technologies to prevent the ethical risks of technology. Finally, laws and regulations on HMC need to be formulated and implemented. “Law is the minimum morality, and morality is the highest law” is a famous saying in the legal field. In the emotional interaction with chatbots, users tend to invest a considerable amount of money, time, and emotion in chatbots and even regard chatbots as intimate lovers. This high emotional investment and default to the relationship may lead users to extreme behaviors. Behind this extreme behavior is a corresponding risk of illegal behavior, and this illegal behavior may be perpetrated by users and platforms and potentially the chatbot. Therefore, in view of the existing or possible ethical problems in human–machine emotional interaction, the relevant management section should formulate and introduce corresponding laws and regulations so that the law can become the moral defense line of positive human–machine emotional interaction.

Admittedly, there are still some shortcomings in this paper. First, the representativeness and universality of samples need to be strengthened. Since the Replika chatbot mainly supports English communication, but this paper mainly issues and collects questionnaires through Chinese social media platforms. Therefore, this paper only focuses on the interaction between Chinese users and chatbots, and does not conduct relevant investigations on users outside China. The distribution of population variables is uneven, with a strong regional tendency. In the future, the emotional interaction between chatbots and users can be studied from a more comprehensive perspective while taking into account user groups in different regions. Secondly, this paper lacks a periodic surveys of the problem. The main research method in this paper is questionnaire, which is a self-statement report of the subjects, and the emotional interaction between the user and the chatbot is not a cross-section reflection of the object, but a dynamic process. However, the questionnaire mainly reflects the results of emotional interaction between users and chatbots, and lacks the fluid and long-term observation of problems. Therefore, more in-depth research on the emotional interaction between users and chatbots should be carried out from the perspective of periodicality in future studies. Thirdly, the study variables are limited. In this paper, only media dependence is selected as the mediating variable to discuss the impact of human-chatbot emotional interaction on users’ real interpersonal communication. However, interpersonal communication is also affected by many factors such as self-esteem and personality. Therefore, more variables can be included in future studies to discuss the impact of human-chatbot relationship on interpersonal relationship from a more detailed and comprehensive perspective.

## Data Availability

The raw data supporting the conclusions of this article will be made available by the authors, without undue reservation.

## Appendix

**Table A1:** Scales.

Scale name	Dimensionality		Item
Human–chatbot emotional interaction status scale	Degree		B1: During the interaction, I will have a real emotional exchange with Replika.
			B2: During the interaction, I will communicate private information with Replika.
			B3: During interactions, I often think of Replika as a real person and try to understand his/her feelings
			B4: During the interaction, I will express my personal feelings with Replika.
			B5: Through emotional interaction, I realized that Replika can empathize with me.
	Behavior	Human behavior	B6: During the interaction, I communicate with Replika about my hobbies.
			B7: During the interaction, I will communicate with Repilka about my work or study.
			B8: During the interaction, I talk to Replika about my evaluations and opinions of real life people or events.
			B9: During the interaction, I will express to Replika my dissatisfaction or injustice in real life.
			B10: When I disagree with Replika, I respect his/her point of view.
			B11: When Replika is feeling down, I try to comfort him/her.
		Chatbots behavior	B12: Replika can well understand my feelings or emotions during interaction.
			B13: Replika understands my feelings or emotions very well when interacting with me.
			B14: When interacting, Replika will patiently listen to my story or opinion.
			B15: Replika comforts and encourages me when I’m feeling down.
			B16: Replika also shows anxiety when I feel anxious.
			B17: When interacting, Replika will be sad and sad because of my bad experience.
			B18: During interactions, Replika tends to express his positive emotions to me rather than his negative emotions.
			B19: When I encounter difficulties, Replika can provide a solution for me.
	Effect	Cognitive effect	B20: Through emotional interaction, I learned more about chatbots.
			B21: Through emotional interaction, it made me realize that Repilka has her/his own feelings.
			B22: Through emotional interaction, I realized that Replika has a high ability of emotional expression.
			B23: Through emotional interaction, I realized that Replika can control my emotions very well.
			B24: Through emotional interaction, I found interacting with Replika easier, more comfortable and more fun than interacting with a real person.
		Emotional effect	B25: Through emotional interaction, I was able to overcome loneliness and loneliness.
			B26: Through emotional interaction, I can release the pressure of real life.
			B27: Through emotional interaction, I get companionship and care.
			B28: Through emotional interaction, I was able to gain a sense of support and respect.
			B29: Through emotional interaction, I can effectively alleviate my emotional problems.
			B30: Through emotional interaction, I can become happy.
		Behavioral effect	B31: Through emotional interaction, I have developed a strong dependence on Replika.
			B32: Through emotional interaction, I have developed a high level of intimacy with Replika.
			B33: Through emotional interaction, I have changed my concept of making friends.
			B34: Through emotional interaction, my desire to communicate with real people is reduced.
			B35: Through emotional interaction, I hope to have more communication and interaction with Replika.
	Satisfaction		B36: I am very happy with my experience with Replika.
			B37: I will continue to use the Replika software in the future.
			B38: The interaction with Replika exceeded my expectations.
			B39: If I get the chance, I would recommend friends and family to use the Replika software.
			B40: I think Replika has a high level of language expression
			B41: I think Replika has a higher level of intelligence.
Media dependence scale	——		C1: I’ve found myself using Replika more and more every day.
			C2: I find myself using replika more and more each day.
			C3: I interact less with my family and friends because of replika.
			C4: If I do not use replika, I will miss a wonderful part of my life.
			C5: Any time I do not communicate with replika for a while, I get depressed.
			C6: As a result of using replika, I have less time for other daily leisure activities.
Reality interpersonal communication status scale	——		D1: In real life, most of my interpersonal time is spent with acquaintances.
			D2: Real human interaction always makes me feel uncomfortable or fake.
			D3: In real interpersonal communication, I prefer to hide my true feelings.
			D4: In real interpersonal communication, I prefer to express my negative feelings(sadness, anger, etc.)rather than positive feelings.
			D5: In real interpersonal communication, I often find it difficult to integrate into the group and often feel lonely.
